# Correspondence from Paris

**Published:** 1848-10-01

**Authors:** 


### CORRESPONDENCE FROM PARIS.

THE causes which I assigned in my last letter for the meagre details which I could offer of the state of psychological science unfortunately still exist. Not only here has the contemplation of the works of nature ceased, but in Italy and in Germany. At the commencement of the present year, the formation of a Society *Medico Psychologique* was announced. On Saturday, the 18th of December, 1847, the institution was constituted, its laws formed, and the catalogue of psychologists, of moralists, of philosophers, of jurisconsults, who had determined to join it, was ready for publication; but such, alas! has been the melancholy condition of the country, that for the present the idea is completely abandoned, and one of the best means for the study of men in this moral and intellectual state is postponed for an unlimited period. Such, also, has been the impossibility of pursuing the usual track, that the number of "*Annales Medico Psychologiques,*" which should have been brought out in May last, has but very lately appeared, and this, probably, is the last time that we shall glance over its valuable pages, from which we have gleaned so much useful and important information. The present state of affairs sufficiently accounts for its possessing less valuable matter than on former occasions; and though it never can cease to be interesting, it has less variety than usual: the most important article is that on Paralysis Pellagrosa, by Dr. Baillarger, which appeared in the last number of your Journal. There is a case, by Dr. Poilhoux, of Castellane, in the Lower Alps, that has already been the subject of much excitement in the part of France in which it occurred; but it is related with so much clearness and judgment, that it has received additional value. It is of some consequence in legal medicine. Two families, composed of fifteen individuals, suddenly rushed from their houses, in a state of nudity, to the parish church, during divine service; they exhibited themselves in the most indecent manner, and were guilty of acts incomprehensible in a well-regulated state of society, and of which the good taste of English medical literature forbids even the description. The following day, they renewed the same disgusting scene, with the exception that, instead of being in a state of perfect nudity, they were covered with some small quantity of linen; they ran through the streets to the public place, vociferating, singing, shouting psalms to the praise of St. Francis. Two days before this strange spectacle, the father of one of the families had been suddenly struck with a disease of a singular character, and was thought to be in a state of complete agony; his body was stiff, his legs and arms contracted, his head fixed immovable on the bed, to which he declared he was bound by a supernatural power. The clergyman of the parish was sent for to give him the last consolations of religion, but he loaded him with reproaches, accusing him of giving him a five-franc piece on which was an effigy of the Republic, and to this he ascribed all his sufferings. After the departure of the clergyman, the sick man was again distressed by the apparition of an insect of a black colour, covered with horns, larger than a scorpion, from whose annoyances he could not escape without burning the bed, the furniture, and every article where the insect could hide itself. The police hearing of the singular occurrence, committed to prison the two fathers and the two mothers, with the elder children. The defence set up was, that at the moment they committed the acts ascribed to them, they were in a state of madness, that they suddenly obeyed an irresistible power within, which completely controlled their movements and urged them on, without their own consent, to commit the indecent acts for which they were reproached. A consultation of medical men was called, who gave it as their unanimous opinion, after frequent examination of the inculpated parties, that they were in a state of mental health; they stated that it could not have been from witchcraft, as these persons alleged that they were thus seized, as, owing to the enlightened state of mankind, it was impossible that such a belief could exist; that it could not have been from the sudden outbreak of an epidemic disease, as individuals living close to them had undergone no similar seizure; that no symptoms of any drug having been taken existed; that the reason would not suddenly yield to the simulation of madness so indecently exhibited in young persons was the only admissible plea for supposing that they were insane; but under the circumstance of their having selected Sunday to give the greater publicity to their extravagant conduct, and their apparently obeying certain orders which emanated from one of the party, which would not have been the case in a band of lunatics; therefore, under all the circumstances, they concluded that the madness was feigned, that the heads of the families had concerted the scheme, and that the younger branches had most probably been induced to obey by menaces. These conclusions were confirmed by the tribunal at Castellane, after a long inquiry and a careful examination of witnesses. From their evidence, it was shown that the poor children had been drilled previously; that they had exclaimed that if they did not obey their parents they would be beat; that the individual who had feigned madness was in dread of a trial in which he had a personal interest. The tribunal condemned the parents to imprisonment, and the Court of Appeal confirmed the sentence.

Scarcely do the volumes of the *Causes Célèbres* contain a more singular series of extraordinary facts than come out; the editorial observations are generally well written; and the allusions to the many instances which have occurred of epidemic madness exhibit the reading and information necessary for the conduct of such a journal. There is the report of the trial of a woman, named Maria Madeleine Langlors, who poisoned a young girl with a cake of arsenic. She was acquitted on the ground of insanity. The case affords matter for much reflection, and shows how much more readily a French court of law listens to a plea of madness than does an English one. Upon the evidence, as it appears in the work before us, no English jury would have given a similar verdict.

No other work has issued from the press upon psychological medicine since my last, and there have been no papers of any interest connected with the subject read before any of the learned societies. In the sitting of the Academie de Médécine, on the 23rd of May, M. Rochoux read, in his own name and that of M. Falret, a report upon a paper drawn up by M. Pereire, of Bordeaux, upon Epilepsy, treated by *L'Arteriotome épicranienne.* It consists of obliterating, by subcutaneous incisions at their origin, the different arterial branches, from which the ramifications spread upon the pericranium in numerous capillary vessels, and establish anastomoses with those of the brain, after having traversed the cranium and its membranes. This obliteration prevents the blood, not only from arriving at the encephalon in sufficient quantity to produce congestion, but produces a kind of atrophy which prevents the return of the fits impossible. This theory appears to M. Rochoux inadmissible; he does not think that congestion determined to the encephalon is necessary for the production of epilepsy, nor does he think that the remedy could be employed to prevent the development of the collateral arteries and their anastomoses.

M. Belhomme has commenced an interesting inquiry into the influence of political commotions and public events in the development of insanity. He has read a memorial, which will, doubtless, in quiet times, be enlarged; and such are the opportunities which the present period affords, that there is little hesitation in affirming that the subject will have more light thrown upon it in six months than it has had in the last thirty years. I have had illustrations, even in my own limited circle of seeing the effects, whilst those who have under their control the great establishments of the country, must daily see instances of the most distressing character—some arising from sudden fright, others from loss of fortune, and the overthrow of all their hopes; and it must be remembered that there are yet calamities in store, for no one who watches the march of events can conceal from himself that, before society returns to its wonted state, there must be great individual as well as public suffering.

Dr. Baillarger has commenced a volume entitled, "Recherches sur l'Anatomie, la Physiologie, et la Pathologie du Systeme Nerveux." A portion has issued from the press, and promises to be, as everything is that issues from his pen, a valuable contribution to medical science; but, alas! the fearful times have put a stop to his intentions, and an unfinished volume is the result; there are some statistical tables on the hereditary nature of mental disease, which throw a vast quantity of light upon a subject not yet sufficiently explored. He has also taken an admirable survey of the means employed to administer by forcible means nutrition to the insane. There are many of what may be called sketches of various states and stages of mental aberration, which, when filled out, will materially add to our stock of knowledge. Indeed, this indefatigable observer is doing more for science than any one, with the exception, probably, of Jacobi, the enlightened physician to the Lunatic Asylum at Siegbourg. This venerable man is the chief of the German school of psychiatric experience. Arrived at an age when most men abandon their labours for literary ease, he continues, after a life spent in doing good, to write and to instruct. His last work, "Die Hauptformen der Seelenstorungen in ihrem Beziehung zur Heilkunde, nach der Beobachtung geschildert," which may be translated, "Upon the principal Forms of Disturbance of the Mind in relation with the Art of curing them," deserves to be translated into every language. It first appeared somewhat more than three years since, and a new edition is preparing, which will contain such improvements as still further experience of the art of curing suggests; the conclusions that he has arrived at, after comparing cases in his own practice with those related by the most eminent men who have written, are given with great ability, and those which more embrace his views upon the state of the circulation of the blood, carry with them intense interest. M. Andrieu, Professor at the School of Medicine at Amiens, announced, some short time since, that there was a cataleptic epidemy at the Maison de Refuge de bon Pasteur, in that city. There are in that establishment 90 penitent recluses, and 12 nuns. Two sisters and more than 20 penitents have been affected; a memoir was read before the Academie de Médecin, and M. Andrieu is about to publish the cases. The singular fact of the remarkable depression of temperature in men and animals under the influence of ether and chloroform is leading to some curious experiments, from which their effects upon febrile and inflammatory conditions of the system may be ascertained. This depression is evidently much more considerable upon animals submitted to the vapour of ether than to the inhalation of chloroform, the effect being precisely the same if introduced by the rectum as by the lungs: it seems to be established now that chloroform, in consequence of the rapidity of its action and the variable duration of the state of anasthesia which it produces, should be reserved for operations of short duration, and that ether should be preferred in cases of long and serious operation. This opinion, when brought forward by M. Bouisson, was objected to by Velpeau, whose opinion it was that, under all circumstances, chloroform should be substituted for ether. The researches of Roux and of Gerardin are certainly in favour of chloroform. In epilepsy all hopes of its utility seem to be abandoned, since the cases of Dr. Moreau, which seem to prove that it is not altogether without danger. The hachisch, grown at Algiers, has been the subject of some experiments.—A paper read by M. de Courtone has attracted much attention: he has obtained a substance, to which he applies the name of cannabine, from which he anticipates good results in the relief of mental disease; he compares it with the cannabis, grown in France, which gives a resin less active. M. Gastinel, of Cairo, lays claim to the discovery of this principle, which he says he originally obtained, after a long chemical analysis.—M. Bouillaud has again brought forward his theory, that the sense of language and the co-ordinate principle of the movement made to produce speech reside in the anterior lobe of the brain. Early as 1839, he entertained this opinion, and discussed the reasons that led him to entertain it, and during the present session he has again taken up the point. At the discussion, he offered a reward of five hundred francs to any one who would produce a well-authorized case, however remote, where there was a deep-seated alteration of structure of the lobes of the brain without producing a corresponding change in the articulation of sound. M. Rochoux stated an instance which fell under the notice of M. Baillarger, where an individual received a wound in the frontal region, in consequence of the explosion of a mine; there was a "*contusion profonde*" of the two anterior lobes, yet he did not lose the power of speech, and could, during the two days he lived, give an account of his state; he also alluded to a case of a talkative barber, who had met with an accident, which did not prevent his continuing his babble. The discussion has not terminated to the satisfaction of either of the parties, both adhering to their own opinion.—M. Poggiale, the Professor of Chemistry, has announced the discovery of a substance more powerful than either chloroform or ether, to which he attaches the name of adelhide. It is obtained by the distillation of sulphuric acid, water, alcohol, and peroxide of manganese, and rectifying the condensed liquor with chloride of calcium. Its odour is exceedingly strong, and this only militates against its frequent employment.—There is expected to be, shortly, an animated discussion in the Academie, on a paper which was read last January, on madness observed at Vannes since the execution of the ministerial ordonnance in 1839, which prescribes absolute silence day and night; the subject was taken up by Ferrus, Baillarger, Dubois, Macquart, and Londe, but the further consideration was deferred until the printing of the report. Happy shall we be when we can return to our former paths, and find still ardent minds to follow the beaten track. We have lost several distinguished physicians, who have chosen the highways of politics, which seldom lead to any but momentary triumph, instead of the bye-paths of science, which most generally conduct, ultimately, if not to high honours and wealth, to peace of mind and the tranquillity which renders the last days of man calm and happy, waiting, with resignation, for rewards elsewhere.

September 20th.

